# COVID-19 Symptoms by Variant Period in the North Carolina COVID-19 Community Research Partnership, North Carolina, USA

**DOI:** 10.3201/eid2901.221111

**Published:** 2023-01

**Authors:** Michael E. DeWitt, Ashley H. Tjaden, David Herrington, John Schieffelin, Michael Gibbs, William S. Weintraub, John W. Sanders, Sharon L. Edelstein, on behalf of the COVID-19 Community Research Partnership

**Affiliations:** Wake Forest University School of Medicine, Winston-Salem, North Carolina, USA (M.E. DeWitt, D. Herrington, J.W. Sanders);; The George Washington University Milken Institute of Public Health, Rockville, Maryland, USA (A.H. Tjaden, S.L. Edelstein);; Tulane School of Medicine, New Orleans, Louisiana, USA (J.S. Schieffelin);; Atrium Health, Charlotte, North Carolina, USA (M.A. Gibbs);; MedStar Health, Columbia, Maryland, USA (W.S. Weintraub);; Georgetown University, Washington, DC, USA (W.S. Weintraub)

**Keywords:** COVID-19, SARS-CoV-2, coronavirus disease, severe acute respiratory syndrome coronavirus 2, respiratory infections, zoonoses, viruses, symptoms, surveillance, North Carolina, United States

## Abstract

In North Carolina, USA, the SARS-CoV-2 Omicron variant was associated with changing symptomology in daily surveys, including increasing rates of self-reported cough and sore throat and decreased rates of loss of taste and smell. Compared with the pre-Delta period, Delta and Omicron (pre-BA.4/BA.5) variant periods were associated with shorter symptom duration.

The evolution of SARS-CoV-2 during the COVID-19 pandemic has raised interest in evolving disease manifestation and associated severity since early reports of its emergence in December 2019 (*1*). As SARS-CoV-2 variants have evolved, studies have focused on the differences in hospitalizations and deaths (*2*,*3*). Although case reports have described changes in symptoms, they are limited in scope and duration of follow-up (*4*–*8*). Moreover, because these retrospective case investigations are often event based, separating novel symptoms from preinfection symptoms is subject to recency bias (*9*), and does not establish a true distribution of these symptoms, unlike prospective syndromic surveillance. The purpose of this study was to describe the evolution of COVID-19 symptoms and their duration during each variant wave in the North Carolina COVID-19 Community Research Partnership (NC-CCRP), a multisite longitudinal symptom and serosurveillance study in North Carolina, USA, that included results from an electronic daily symptom survey regardless of infection status.

## The Study

**Figure 1:**
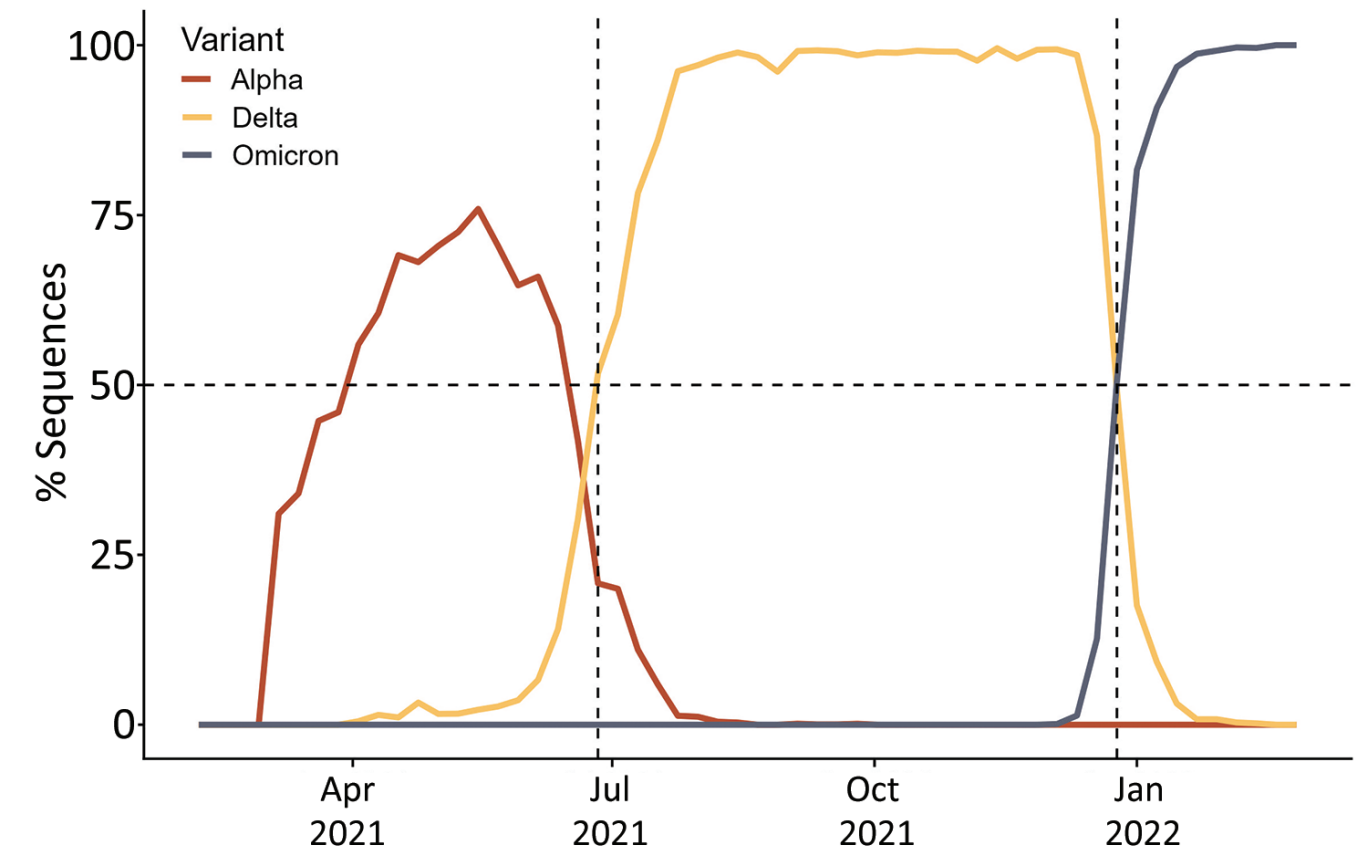
Overview of COVID-19 waves by variant in North Carolina, USA. We defined pre-Delta as an infection before June 26, 2021. Delta was the predominant variant during June 26–December 25, 2021, after which Omicron became dominant. Data were provided by North Carolina Department of Health and Human Services.

The NC-CCRP is one of the largest and longest running syndromic surveillance surveys of a convenience cohort in the United States. In the study, a total of 37,820 adult participants completed daily health and symptom logs during April 2020–April 2022 and captured 5,480 self-reported COVID-19 infections (*10*). Adults >18 years of age were recruited from the patient populations served by healthcare systems at 6 North Carolina sites via direct email outreach. Participants received a brief daily electronic survey by text or email to answer questions about COVID-19 exposures, symptoms, test results, receipt of vaccination, and risk behaviors. We obtained demographic information and healthcare worker occupation at baseline. Participants provided informed consent electronically. We defined variant periods as pre-Delta, Delta, and Omicron (pre-BA.4/BA.5) based on variant predominance in North Carolina (Figure 1). We defined symptomatic COVID-19 as the presence of >1 new symptom 2 weeks before or after the date of a self-reported positive viral test. A new symptom occurred if the symptom was not present in the 7 days before the report date. We defined reinfection as a positive test result >90 days after a previous positive test.

We estimated the duration of symptoms using a linear model fit to those persons who had a symptomatic episode during their infection where the predominant variant during infection was the independent variable. We included adjustments for participant sex, vaccination and booster status at infection, prior infection status, and participant age as potential confounding factors. We generated estimated marginal means to understand the pairwise comparisons between different variant waves. We analyzed symptom frequency and duration for the proportion of participants reporting each symptom and the duration of symptoms occurring >1 time during the episode. We used Fisher exact test or χ^2^ test to determine symptom frequency between the variant waves and performed Kruskal-Wallis rank sum tests to identify differences in symptom duration. We used false-discovery rate corrections to adjust for multiple comparisons in which α = 0.05. We conducted all analysis in R version 4.1.3 (The R Project for Statistical Computing, https://www.r-project.org).

Self-reported reinfections increased from 0.4% during pre-Delta and 2.9% during Delta to 6.3% during Omicron (Table 1). During the pre-Delta period, most infections were among persons not yet fully vaccinated (97.5%). During the Delta and Omicron periods, nearly all participants were fully vaccinated at the time of infection, 84% Delta and 93% Omicron (Table 1), which corresponded to the higher rates of vaccination in the cohort during these periods. Survey participation rate declined from 37,711 participants during the pre-Delta period to 19,189 participants during the Omicron period; the median age increased from 49 to 55 years of age (Table 1).

**Table 1:** Overview of demographics and key characteristics of study population in the North Carolina COVID-19 Community Research Partnership study on COVID-19 reinfection, North Carolina, USA*

Characteristic	Pre-Delta, n = 37,711	Delta, n = 24,664	Omicron, n = 19,189
Median age, y (IQR)	49 (37–62)	53 (41–64)	55 (43–65)
Sex			
F	26,001 (69)	17,156 (70)	13,233 (69)
M	11,710 (31)	7,508 (30)	5,956 (31)
Race			
Asian	727 (1.9)	404 (1.6)	296 (1.5)
Black or African American	2,122 (5.6)	1,205 (4.9)	870 (4.5)
White	32,946 (87)	21,985 (89)	17,218 (90)
Other	1,916 (5.1)	1,070 (4.3)	805 (4.2)
Ethnicity			
Other	3,245 (8.6)	1,748 (7.1)	1,266 (6.6)
Hispanic or Latino	942 (2.5)	562 (2.3)	442 (2.3)
Mixed ethnicity	500 (1.3)	317 (1.3)	239 (1.2)
Not Hispanic/Latino	33,024 (88)	22,037 (89)	17,242 (90)
Healthcare worker	10,488 (28)	6,914 (28)	5,077 (26)
Site			
Atrium	8,362 (22)	6,112 (25)	4,983 (26)
Campbell	744 (2.0)	450 (1.8)	259 (1.3)
New Hanover	716 (1.9)	540 (2.2)	0 (0)
Vidant	1,367 (3.6)	692 (2.8)	0 (0)
Wake Forest	23,475 (62)	14,628 (59)	12,122 (63)
Wake Med	3,047 (8.1)	2,242 (9.1)	1,825 (9.5)
Median no. days reporting during wave (IQR)	169 (62–326)	129 (56–167)	47 (22–57)
Vaccination status			
Fully vaccinated by beginning of wave	1,149 (3.0)	21,772 (88)	18,092 (94)
Fully vaccinated by end of wave†	24,264 (64)	22,597 (92)	18,112 (94)
Boosted by beginning of wave‡	0	122 (0.5)	14,595 (76)
Boosted by end of wave‡	6 (<0.1)	15,623 (63)	15,348 (80)
COVID-19 positive test§	1,908 (5.1)	1,472 (6.0)	2,090 (11)
Reinfection¶	8 (0.4)	43 (2.9)	132 (6.3)
Vaccine breakthrough infection	47 (2.5)	1,233 (84)	1,936 (93)
Booster breakthrough infection	0	360 (24)	1,444 (69)

*Values are no. (%) except as indicated. IQR, interquartile range. †>14 days after receipt of first dose if first dose is Johnson & Johnson/Janssen (https://www.jnj.com); >14 days after date of receipt of second dose if first dose is Pfizer-BioNTech (https://www.pfizer.com), Moderna (https://www.modernatx.com), other, or missing. ‡Self-reported receipt of a second dose >60 days following an initial Janssen vaccination or reported a third dose >60 days after the receipt of 2 doses of Pfizer/Moderna/other/missing. §Self-reported. ¶ Self-reported positive test (COVID-19 infection (sample by nasal swab, saliva or spit) >90 days after a prior positive test.

**Figure 2:**
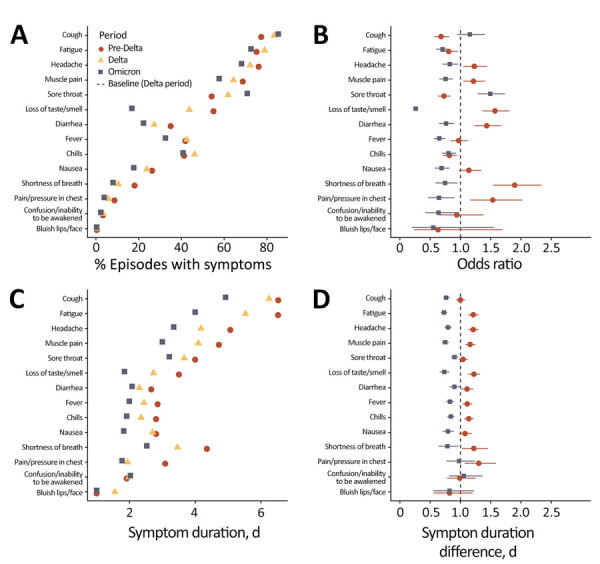
Reported COVID-19 symptoms by variant in the North Carolina COVID-19 Community Research Partnership, North Carolina, USA. A) Proportion of symptomatic persons reporting symptoms; we defined a symptomatic episode as the presence of any new symptoms in the 14-day window, where new means not occurring in the 7 days preceding the first observation of the symptom within the window. B) Odds ratio of reporting a symptom by variant wave using Delta as the baseline. C) Average length of participant-reported symptom duration in a symptomatic infection. D) Difference in symptom duration from Delta-period infections. Error bars represent 95% CI.

Cough was the most frequent self-reported symptom in all waves; it increased from 77% pre-Delta to 85% during Omicron (p = 0.001) (Figure 2, panel A). Sore throat was more common during Omicron (71%), compared with 62% during Delta and 54% during pre-Delta (p<0.001). The largest change in proportion reporting a symptom was loss of taste or smell, which decreased from 55% during pre-Delta to 17% during Omicron (p<0.001). Compared with the pre-Delta period, the Delta (−1.26, 95% CI −1.95 to −0.57) and Omicron periods (−3.82, 95% CI −4.55 to −3.09) were associated with shorter symptom duration (Table 2; Figure 1, panel D). We conducted a sensitivity analysis to examine the effects of different comorbidities; we found that those with autoimmune, pulmonary, and renal diseases and obesity had longer symptom durations when adjusting for variant wave, age, vaccination, prior infection, and sex (Appendix Table 1). We observed no statistically significant interactions between variant period and comorbidity. A sensitivity analysis stratified by those who were and were not fully vaccinated before symptom onset found no appreciable difference in symptomology or length of symptoms than in the whole cohort combined (Appendix Tables 3–4). When we stratified results by variant, vaccination during Delta was associated with lower odds of reporting all individual symptoms except fatigue and sore throat (Appendix Figure 1).

**Table 2:** Symptom duration among participants reporting symptoms by variant wave in study of COVID-19 infection, North Carolina, USA*

Variant period	Beta (95% CI)†	p value	Marginal mean duration, d (95% CI)‡	
Pre-Delta	NA	NA	9.7 (9.0–10.4)	
Delta	−1.3 (−2.0 to −0.57)	<0.001	8.4 (7.8–9.1)	
Omicron	−3.8 (−4.6 to −3.1)	<0.001	5.9 (5.3–6.4)	

*Data are adjusted for age, vaccination status, boosting, reinfection, and sex. NA, not applicable. †Differences from reference wave (pre-Delta) in the linear model. ‡Marginal mean represents the estimated mean duration of a symptomatic period across all levels of age, vaccination status, boosting, reinfection, and sex.

## Conclusions

Our results identify notable shifts in clinical manifestation and symptomology during the different phases of the COVID-19 pandemic. These findings support accumulating evidence of increasing occurrences of breakthrough infections in vaccinated and boosted participants and growing rates of reinfection commensurate with the rise in prevalence of the Omicron variant in the United States (*2*,*5*,*11*–*13*).

In this longitudinal survey of self-reported symptoms we found that variant waves were associated with differing symptom prevalence and duration. Overall, these findings largely confirm observations that symptomatic Omicron infections resolve faster with less severe symptomology, often resembling an upper respiratory infection without loss of taste and smell; and are less likely to result in hospitalization (*13*,*14*). Our estimates of marginal mean symptom duration and are consistent with Menni et al.; we found average duration of 8.4 (95% CI 7.8–9.1) days for Delta and 5.9 (95% CI 5.3–6.4) days for Omicron infections. Menni et al. found average symptom durations of 8.89 (95% CI 8.61–9.17) days for Delta and 6.87 (95% CI 6.58–7.16) days for Omicron infections in matched analysis of a prospective longitudinal syndromic study in the United Kingdom (*13*). Studies have shown that Omicron spike is associated with higher ACE2 binding affinity and less efficient S1/S2 cleavage than Delta, resulting in lower rates of syncytia formation (*15*). These changes in cellular tropism may explain the observed shift in symptom presentation and decreased symptom duration during Omicron.

A limitation of this study is that survey participant age declined during the study, whereas median age increased. Those who responded in later periods may have been more likely to report symptoms, and thus our estimates may overstate the duration and intensity of symptoms during the Omicron period. Respondents self-selected for participation, and some participants may be more likely to report symptoms than others, which is a limitation in self-reported symptom surveys. The survey also did not explicitly capture the use of at-home antigen tests, which expanded during the Omicron period.

Despite the lack of individual-level genomic sequencing, we detected significant differences in symptom manifestation and duration; our findings indicate that continued longitudinal syndromic surveillance could be an important component in measuring disease presentation, outcomes, and prevalence. As COVID-19 becomes endemic and the immune landscape changes through vaccination and infection, understanding symptomology and clinical presentation will be needed to distinguish SARS-CoV-2 from other viral infections and provide insight into evolving pathology.

Appendix Additional information about COVID-19 symptoms by variant period reported in the North Carolina COVID-19 Community Research Partnership.
