# Supplementary material for: COVID-19 Symptoms by Variant Period in the North Carolina COVID-19 Community Research Partnership, North Carolina, USA
Source: Emerg Infect Dis. 2023 Jan;29(1):207–11. doi: 10.3201/eid2901.221111 (PMC9796200; doi:10.3201/eid2901.221111)
Supplement: Appendix — Additional information about COVID-19 symptoms by variant period reported in the North Carolina COVID-19 Community Research Partnership. [file 22-1111-Techapp-s1.pdf]

Article DOI: https://doi.org/10.3201/eid2901.221111

COVID-19 Symptoms by Variant Period in the North Carolina COVID-19 Community Research Partnership

Appendix

COVID-19 Community Research Partnership

(*Site Principal Investigator)

Wake Forest School of Medicine: Thomas F Wierzba PhD, MPH, MS,* John Walton Sanders, MD, MPH, David Herrington, MD, MHS, Mark A. Espeland, PhD, MA, John Williamson, PharmD, Morgana Mongraw-Chaffin, PhD, MPH, Alain Bertoni, MD, MPH, Martha A. Alexander-Miller, PhD, Paola Castri, MD, PhD, Allison Mathews, PhD, MA, Iqra Munawar, MS, Austin Lyles Seals, MS, Brian Ostasiewski, Christine Ann Pittman Ballard, MPH, Metin Gurcan, PhD, MS, Alexander Ivanov, MD, Giselle Melendez Zapata, MD, Marlena Westcott, PhD, Karen Blinson, Laura Blinson, Mark Mistysyn, Donna Davis, Lynda Doomy, Perrin Henderson, MS, Alicia Jessup, Kimberly Lane, Beverly Levine, PhD, Jessica McCanless, MS, Sharon McDaniel, Kathryn Melius, MS, Christine O'Neill, Angelina Pack, RN, Ritu Rathee, RN, Scott Rushing, Jennifer Sheets, Sandra Soots, RN, Michele Wall, Samantha Wheeler, John White, Lisa Wilkerson, Rebekah Wilson, Kenneth Wilson, Deb Burcombe, Georgia Saylor, Megan Lunn, Karina Ordonez, Ashley O'Steen, MS, Leigh Wagner.

Atrium Health: Michael S. Runyon MD, MPH,* Lewis H. McCurdy MD,* Michael A. Gibbs, MD, Yhenneko J. Taylor, PhD, Lydia Calamari, MD, Hazel Tapp, PhD, Amina Ahmed, MD, Michael Brennan, DDS, Lindsay Munn, PhD, RN, Keerti L. Dantuluri, MD, Timothy Hetherington, MS, Lauren C. Lu, Connell Dunn, Melanie Hogg, MS, CCRA, Andrea Price, Marina Leonidas, Melinda Manning, Whitney Rossman, MS, Frank X. Gohs, MS, Anna Harris, MPH, Jennifer S. Priem, PhD, MA, Pilar Tochiki, Nicole Wellinsky, Crystal Silva, Tom Ludden PhD, Jackeline Hernandez, MD, Kennisha Spencer, Laura McAlister.

MedStar Health Research Institute: William Weintraub MD,* Kristen Miller, DrPH, CPPS,* Chris Washington, Allison Moses, Sarahfaye Dolman, Julissa Zelaya-Portillo, John Erkus, Joseph Blumenthal, Ronald E. Romero Barrientos, Sonita Bennett, Shrenik Shah, Shrey Mathur, Christian Boxley, Paul Kolm, PhD, Ella Franklin, Naheed Ahmed, Moira Larsen.

Tulane: Richard Oberhelman MD,* Joseph Keating PhD,* Patricia Kissinger, PhD, John Schieffelin, MD, Joshua Yukich, PhD, Andrew Beron, MPH, Johanna Teigen, MPH.

University of Maryland School of Medicine: Karen Kotloff MD,* Wilbur H. Chen MD, MS,* DeAnna Friedman-Klabanoff, MD, Andrea A. Berry, MD, Helen Powell, PhD, Lynnee Roane, MS, RN, Reva Datar, MPH, Colleen Reilly.

University of Mississippi: Adolfo Correa MD, PhD,* Bhagyashri Navalkele, MD, Yuan-I Min, PhD, Alexandra Castillo, MPH, Lori Ward, PhD, MS, Robert P. Santos, MD, MSCS, Pramod Anugu, Yan Gao, MPH, Jason Green, Ramona Sandlin, RHIA, Donald Moore, MS, Lemichal Drake, Dorothy Horton, RN, Kendra L. Johnson, MPH, Michael Stover.

Wake Med Health and Hospitals: William H. Lagarde MD,* LaMonica Daniel, BSCR.

New Hanover: Patrick D. Maguire MD,* Charin L. Hanlon, MD, Lynette McFayden, MSN, CCRP, Isaura Rigo, MD, Kelli Hines, BS, Lindsay Smith, BA, Monique Harris, CCRP, Belinda Lissor, AAS, CCRP, Vivian Cook, MA, MPH, Maddy Eversole, BS, Terry Herrin, BS, Dennis Murphy, RN, Lauren Kinney, BS, Polly Diehl, MS, RHIA, Nicholas Abromitis, BS, Tina St. Pierre, BS, Bill Heckman, Denise Evans, Julian March, BA, Ben Whitlock, CPA, MSA, Wendy Moore, BS, AAS, Sarah Arthur, MSW, LCSW, Joseph Conway.

Vidant Health: Thomas R. Gallaher MD,* Mathew Johanson, MHA, CHFP, Sawyer Brown, MHA, Tina Dixon, MPA, Martha Reavis, Shakira Henderson, PhD, DNP, MS, MPH, Michael Zimmer, PhD, Danielle Oliver, Kasheta Jackson, DNP, RN, Monica Menon, MHA, Brandon Bishop, MHA, Rachel Roeth, MHA.

Campbell University School of Osteopathic Medicine: Robin King-Thiele DO,* Terri S. Hamrick PhD,* Abdalla Ihmeidan, MHA, Amy Hinkelman, PhD, Chika Okafor, MD (Cape Fear Valley Medical Center), Regina B. Bray Brown, MD, Amber Brewster, MD, Danius Bouyi, DO, Katrina Lamont, MD, Kazumi Yoshinaga, DO, (Harnett Health System), Poornima Vinod, MD, A. Suman Peela, MD, Giera Denbel, MD, Jason Lo, MD, Mariam Mayet-Khan, DO, Akash Mittal, DO, Reena Motwani, MD, Mohamed Raafat, MD (Southeastern Health System), Evan Schultz, DO, Aderson Joseph, MD, Aalok Parkeh, DO, Dhara Patel, MD, Babar Afridi, DO (Cumberland County Hospital System, Cape Fear Valley).

George Washington University Data Coordinating Center: Diane Uschner PhD,* Sharon L. Edelstein, ScM, Michele Santacatterina, PhD, Greg Strylewicz, PhD, Brian Burke, MS, Mihili Gunaratne, MPH, Meghan Turney, MA, Shirley Qin Zhou, MS, Ashley H Tjaden, MPH, Lida Fette, MS, Asare Buahin, Matthew Bott, Sophia Graziani, Ashvi Soni, MS, Guoqing Diao, PhD, Jone Renteria, MS.

George Washington University Mores Lab: Christopher Mores, PhD, Abigail Porzucek, MS.

Oracle Corporation: Rebecca Laborde, Pranav Acharya.

Sneez LLC: Lucy Guill, MBA, Danielle Lamphier, MBA, Anna Schaefer, MSM, William M. Satterwhite, JD, MD.

Vysnova Partners: Anne McKeague, PhD, Johnathan Ward, MS, Diana P. Naranjo, MA, Nana Darko, MPH, Kimberly Castellon, BS, Ryan Brink, MSCM, Haris Shehzad, MS, Derek Kuprianov, Douglas McGlasson, MBA, Devin Hayes, BS, Sierra Edwards, MS, Stephane Daphnis, MBA, Britnee Todd, BS.

Javara Inc.: Atira Goodwin.

External Advisory Council: Ruth Berkelman, MD, Emory University; Kimberly Hanson, MD, University of Utah; Scott Zeger, PhD, Johns Hopkins University; Cavan Reilly, PhD, University of Minnesota; Kathy Edwards, MD, Vanderbilt University; Helene Gayle, MD MPH, Chicago Community Trust; Stephen Redd.

Appendix Table 1. Estimated effect of comorbidities on duration of COVID-19 symptoms among those expressing symptoms, North Carolina, USA

Comorbidity*	Beta†	95% CI	p value
Autoimmune disease	0.58	0.13–1.04	0.012
Cancer	-0.41	-1.14 to 0.32	0.274
Cardiovascular disease	0.11	-0.3 to 0.52	0.591
Pulmonary disease	0.93	0.38–1.48	<0.001
Obesity	0.61	0.16–1.06	0.008
Diabetes	0.43	-0.2 to 1.07	0.183
Immunocompromised	0.61	-0.71 to 1.92	0.366
Renal disease	1.33	0.05–2.62	0.042

*Adjusted for variant wave, age, vaccination, boosting, prior infection, and sex.
†Difference in symptom duration (days)

Appendix Table 2.: Symptom frequency and duration amongst those unvaccinated against SARS-CoV-2 at time of infection and expressed symptoms

	Rate of occurrence*				Duration of symptoms†			
Symptom	Pre-Delta, N = 1,720	Delta, N = 229	Omicron, N = 137	p value‡	Pre-Delta, N = 1,720	Delta, N = 229	Omicron, N = 137	p value§
Cough	1,329 (77)	200 (87)	110 (80)	0.002	5 (2–10)	5 (2–10)	2 (1–5)	<0.001
Fatigue	1,295 (75)	182 (79)	95 (69)	0.092	5 (2–9)	4 (2–9)	2 (1–4)	<0.001
Headache	1,314 (76)	183 (80)	95 (69)	0.070	4 (2–7)	3 (1–6)	2 (1–4)	<0.001
Muscle pain	1,179 (69)	177 (77)	84 (61)	0.003	3 (2–6)	3 (1–7)	2 (1–3)	<0.001
Sore throat	931 (54)	133 (58)	85 (62)	0.13	3 (1–5)	2 (1–5)	2 (1–3)	<0.001
Loss of taste/smell	949 (55)	126 (55)	24 (18)	<0.001	2 (1–4)	2 (1–4)	1 (1–2)	<0.001
Diarrhea	601 (35)	93 (41)	37 (27)	0.030	2 (1–3)	2 (1–3)	1 (1–2)	0.012
Fever	722 (42)	125 (55)	63 (46)	0.001	2 (1–4)	2 (1–4)	2 (1–2)	0.11
Chills	710 (41)	127 (55)	64 (47)	<0.001	2 (1–3)	2 (1–4)	1 (1–2)	0.005
Nausea	452 (26)	86 (38)	30 (22)	<0.001	2 (1–3)	2 (1–4)	1 (1–2)	0.012
Shortness of breath	310 (18)	49 (21)	14 (10)	0.024	2 (1–5)	2 (1–3)	1 (1–1)	0.016
Pain and pressure in chest	148 (8.6)	25 (11)	8 (5.8)	0.2	1 (1–3)	2 (1–3)	1 (1–1)	0.3
Confused/ inability to awake/arose	56 (3.3)	17 (7.4)	4 (2.9)	0.006	1 (1–2)	1 (1–1)	1 (1–1)	0.4
Bluish lips/face	7 (0.4)	4 (1.7)	1 (0.7)	0.034	1 (1–1)	1 (1–2)	1 (1–1)	0.4

*Values are no. (%) except as indicated.
†Values are median (IQR) except as indicated. IQR, interquartile range.
‡By Pearson χ^2 test, Fisher exact test.
§By Kruskal-Wallis rank sum test.

Appendix Table 3. Symptom frequency and duration amongst those vaccinated, but not boosted, against SARS-CoV-2 at time of infection and expressed symptoms

	Rate of occurrence*				Duration of symptoms, d†			
Characteristic	Pre-Delta, N = 28	Delta, N = 844	Omicron, N = 436	p value‡	Pre-Delta, N = 28	Delta, N = 844	Omicron, N = 436	p value§
Cough	23 (82)	701 (83)	384 (88)	0.051	3 (2–7)	5 (2–9)	3 (1–7)	<0.001
Fatigue	19 (68)	680 (81)	338 (78)	0.14	5 (4–12)	4 (2–8)	3 (1–5)	<0.001
Headache	17 (61)	631 (75)	310 (71)	0.12	3 (2–5)	3 (2–6)	2 (1–4)	<0.001
Muscle pain	21 (75)	546 (65)	293 (67)	0.4	3 (1–6)	3 (2–5)	2 (1–3)	<0.001
Sore throat	16 (57)	494 (59)	299 (69)	0.002	3 (2–7)	3 (1–5)	2 (1–4)	0.016
Loss of taste/smell	12 (43)	443 (52)	95 (22)	<0.001	2 (2–4)	2 (1–3)	1 (1–2)	<0.001
Diarrhea	10 (36)	241 (29)	127 (29)	0.7	2 (1–4)	1 (1–3)	1 (1–2)	0.045
Fever	9 (32)	378 (45)	173 (40)	0.11	2 (1–3)	2 (1–3)	1 (1–2)	<0.001
Chills	10 (36)	422 (50)	232 (53)	0.2	2 (1–6)	2 (1–3)	1 (1–2)	<0.001
Nausea	8 (29)	203 (24)	109 (25)	0.8	2 (1–3)	1 (1–3)	1 (1–2)	0.084
Shortness of breath	6 (21)	84 (10.0)	40 (9.2)	0.12	6 (3–9)	2 (1–4)	1 (1–2)	0.009
pain and pressure in chest	4 (14)	46 (5.5)	23 (5.3)	0.14	4 (1–9)	1 (1–2)	1 (1–2)	0.2
Confused/ inability to awake/arose	2 (7.1)	30 (3.6)	12 (2.8)	0.3	4 (3–4)	1 (1–2)	1 (1–2)	0.058
Bluish lips/face	0 (0)	5 (0.6)	3 (0.7)	>0.9	NA	1 (1–1)	1 (1–1)	

*Values are no. (%) except as indicated.
†Values are median (IQR) except as indicated. IQR, interquartile range.
‡By Pearson χ^2 test, Fisher exact test.
§By Kruskal-Wallis rank sum test.

Appendix Table 4. Estimated effect of reinfection of SARS-CoV-2 on duration of symptoms amongst those with symptoms during the Omicron variant period*

Characteristic	Beta†	95% CI	p value
Reinfection	-0.39	-1.4, 0.64	0.5

*Adjusting for age, vaccination, boosting, and sex
†Difference in symptom duration (days).

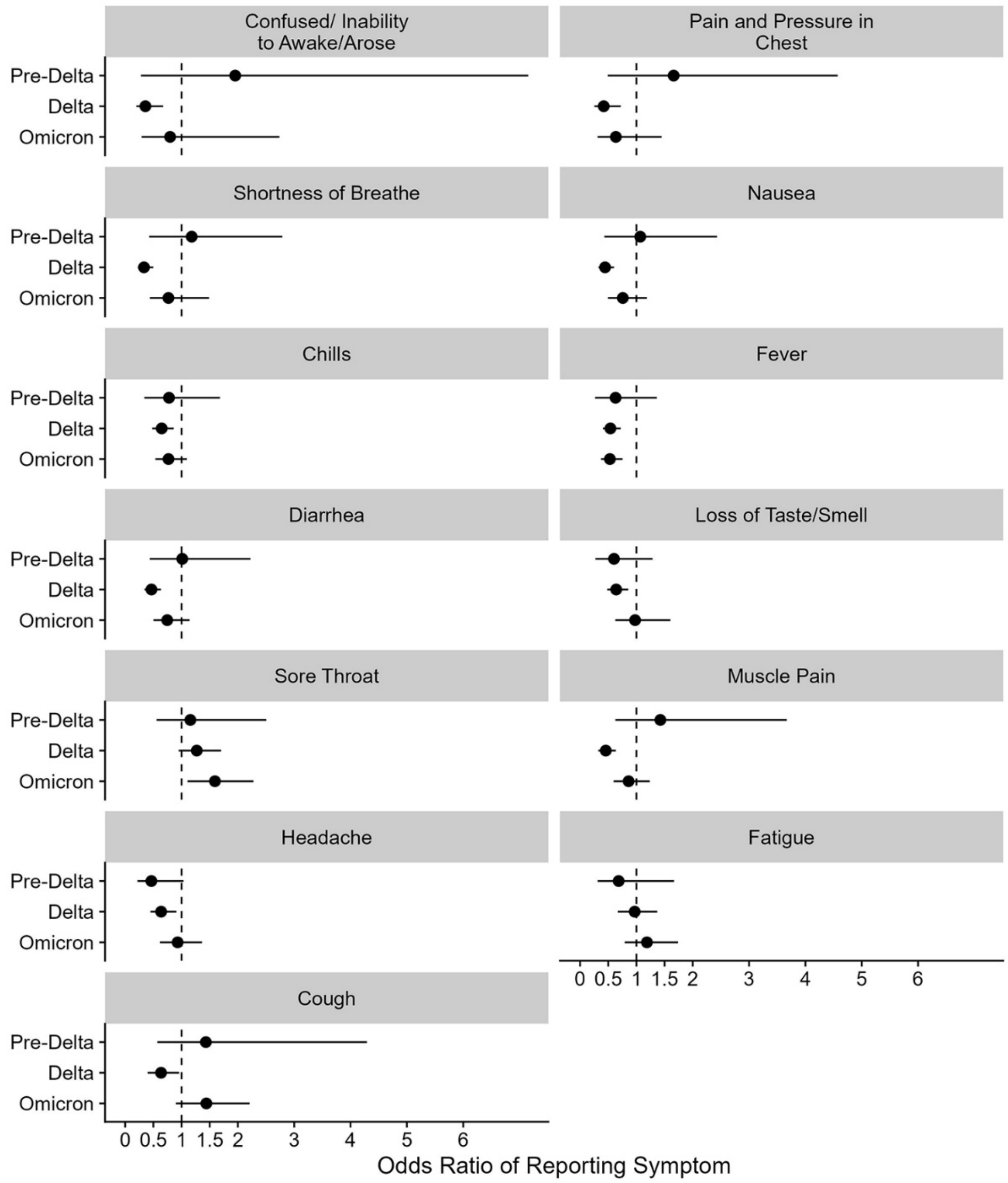

Appendix Figure 1. Odds ratio of reporting a given symptom in those vaccinated at the time of self-reported infection (baseline, unvaccinated) stratified by variant (adjusted for age and sex). Bars indicate 95% Bayesian credible intervals. Dashed line represents parity with unvaccinated persons.

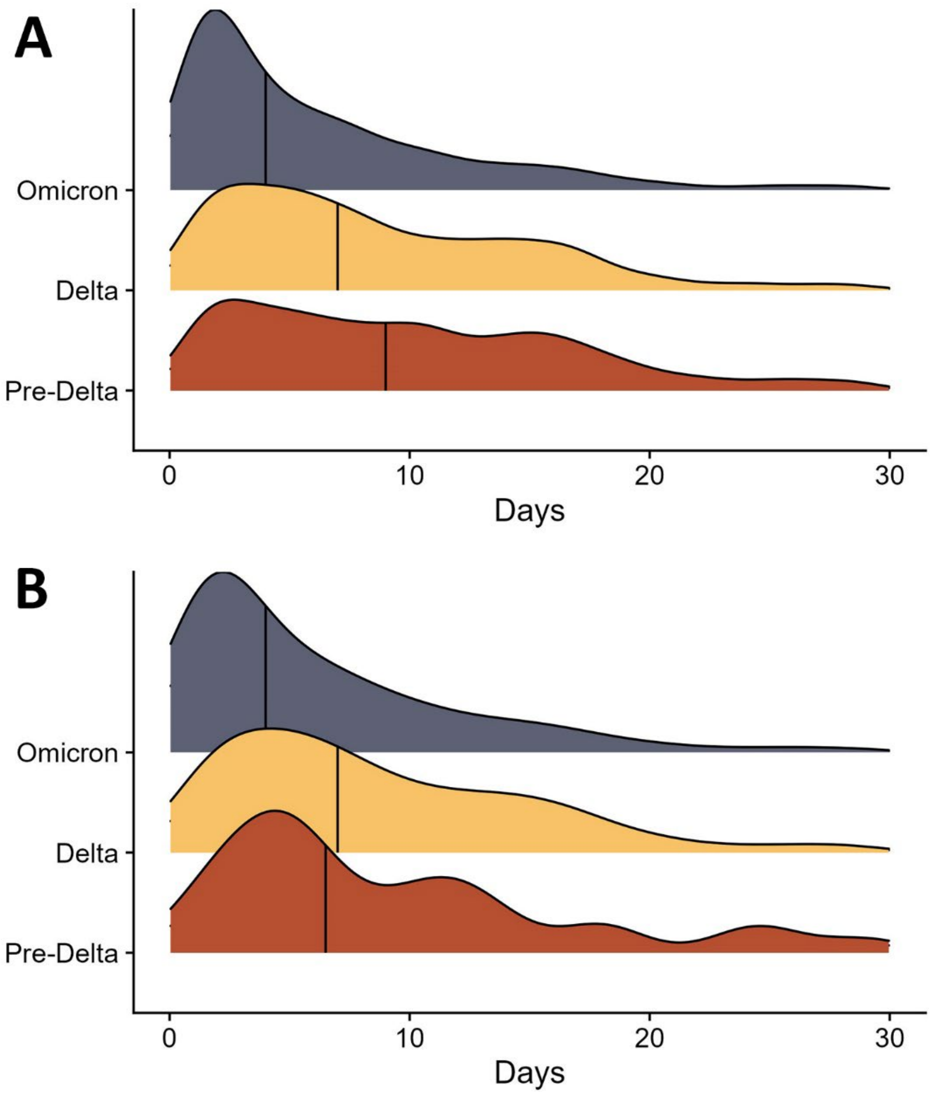

Appendix Figure 2. Kernel density estimates for symptom duration by variant for those who had symptoms, amongst the vaccinated (A) and unvaccinated (B). Vertical line represents the median symptom duration for each stratified variant group.
